# New insights into the mycobacterial PE and PPE proteins provide a framework for future research

**DOI:** 10.1111/mmi.14409

**Published:** 2019-11-24

**Authors:** Louis S. Ates

**Affiliations:** ^1^ Department of Experimental Immunology Amsterdam Infection & Immunity Institute Amsterdam UMC University of Amsterdam Meibergdreef 9 Amsterdam the Netherlands

## Abstract

The PE and PPE proteins of *Mycobacterium tuberculosis* have been studied with great interest since their discovery. Named after the conserved proline (P) and glutamic acid (E) residues in their N‐terminal domains, these proteins are postulated to perform wide‐ranging roles in virulence and immune modulation. However, technical challenges in studying these proteins and their encoding genes have hampered the elucidation of molecular mechanisms and leave many open questions regarding the biological functions mediated by these proteins. Here, I review the shared and unique characteristics of PE and PPE proteins from a molecular perspective linking this information to their functions in mycobacterial virulence. I discuss how the different subgroups (PE_PGRS, PPE‐PPW, PPE‐SVP and PPE‐MPTR) are defined and why this classification of paramount importance to understand the PE and PPE proteins as individuals and or groups. The goal of this MicroReview is to summarize and structure the existing information on this gene family into a simplified framework of thinking about PE and PPE proteins and genes. Thereby, I hope to provide helpful starting points in studying these genes and proteins for researchers with different backgrounds. This has particular implications for the design and monitoring of novel vaccine candidates and in understanding the evolution of the *M. tuberculosis* complex.

## Graphical abstract

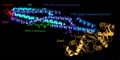

## Introduction

One of the most striking findings of the *Mycobacterium tuberculosis* genome sequencing project was the identification of a family of genes, called *pe* and *ppe* genes, covering up to 10% of the *M. tuberculosis* genome (Cole *et al.*, 1998; Fishbein *et al.*, 2015). The PE and PPE proteins are named after the conserved proline (P) and glutamic acid (E) residues in their N‐terminal sequences and have intrigued researchers since their discovery. However, although significant advances have been made in understanding these proteins, progress has been hampered by technological challenges associated with studying PE and PPE proteins and genes. These challenges include the high *gc*‐content of *pe* and *ppe* genes, which can range up to 80%, making sequencing, alignment and cloning of these genes an ordeal (Hermans *et al.*, 1992; Poulet and Cole, 1995; Cole *et al.*, 1998). This problem is aggravated by many gene duplications and repetitive sequences that distinguish certain subgroups of *pe and ppe* genes (Gey van Pittius *et al.*, 2006; McEvoy *et al.*, 2012; Copin *et al.*, 2014). Because of the challenges associated with mapping *pe*/*ppe* reads to mycobacterial reference genomes, these genes have generally been excluded from frequently used bioinformatic pipelines and have remained relatively understudied with genome‐based techniques (Coll *et al.*, 2014; Phelan *et al.*, 2016; Meehan *et al.*, 2019). Similarly, mass spectrometry of PE and PPE proteins is hampered by a paucity of trypsin cleavage sites and high homology between and within PE and PPE proteins (Banu *et al.*, 2002; Schubert *et al.*, 2013; Ates *et al.*, 2015). However, emerging techniques, such as long‐read sequencing methods (e.g. Nanopore and Pacific Biosciences technologies) and novel biochemical insights into these proteins, may pave the way for new discoveries and improve understanding of the biology of these proteins (Ates *et al.*, 2015; 2018b; Rodríguez *et al.*, 2015; Pandey *et al.*, 2018).

When discussing PE and PPE proteins and their functions, it is paramount to consider their subcellular localization. Some PPE proteins are thought to function as outer membrane nutrient transport proteins and would therefore need to be localized in the mycobacterial outer membrane to perform their function (Ates *et al.*, 2015; Tufariello *et al.*, 2016; Mitra *et al.*, 2017). Other PE and PPE proteins are described to play roles in host–pathogen interaction or immune evasion (Sampson, 2011; Saini *et al.*, 2016). To interact directly with the host cell, the protein needs to be either surface associated and/or secreted in soluble form. In all these cases, the PE and PPE proteins need to be first transported over the bacterial inner membranes, by their cognate TypeVII secretion systems ESX‐1, ESX‐3 and ESX‐5 (Bitter *et al.*, 2009; Houben *et al.*, 2012b; Gröschel *et al.*, 2016; Beckham *et al.*, 2017). For clarity, in this review ‘secretion’ is defined as protein transport over the inner membrane by a TypeVII secretion system.

Due to the methodological obstacles, the precise subcellular localization of PE and PPE proteins is understudied and remains uncertain in many cases. Despite some progress to isolate mycobacterial outer membrane proteins, no single method has been truly standardized or widely adopted (Abdallah *et al.*, 2006; 2009; Sani *et al.*, 2010; van der Woude *et al.*, 2013; Danilchanka *et al.*, 2014; Ates *et al.*, 2015; Fishbein *et al.*, 2015; Mitra *et al.*, 2017). Similarly, different methods of secretion analysis can come to different results depending on timescale, growth medium and the detection method used. Finally, many studies investigating PE and PPE proteins use *Mycobacterium smegmatis* as a model organism, which has no functional ESX‐5 system and is therefore unable to solubly express or translocate the majority of PE and PPE proteins (Abdallah *et al.*, 2007; Sampson, 2011; Gröschel *et al.*, 2016; Beckham *et al.*, 2017).

In spite of all these methodological challenges, perhaps the biggest hurdle for researchers attempting to study PE and PPE proteins is to make sense of the vast body of literature. In this review, I aim to provide a framework to study PE and PPE proteins from a molecular biology perspective by establishing their common characteristics while highlighting the importance of classifying PE and PPE proteins within subgroups (Gey van Pittius *et al.*, 2006). This framework is used to discuss recent progress in understanding of the role of PE and PPE proteins in the pathogenesis, immune recognition and evolution of the *M. tuberculosis* complex (MTBC).

## General features of PE and PPE proteins

To study any protein of interest, it is important to understand the features that define its function. A first step in studying PE and PPE proteins is, therefore, to divide the protein of interest into their conserved PE or PPE domains and the highly variable C‐terminal domains. Although the function of PE and PPE proteins remains mostly unknown, molecular and biochemical studies have greatly improved our understanding of the PE and PPE domains (Strong *et al.*, 2006; Abdallah *et al.*, 2009; Sayes *et al.*, 2012; Ekiert and Cox, 2014; Korotkova *et al.*, 2014). In this section, I will first discuss these general features of the PE and PPE domains before discussing the more variable C‐terminal domains.

**Figure 1:**
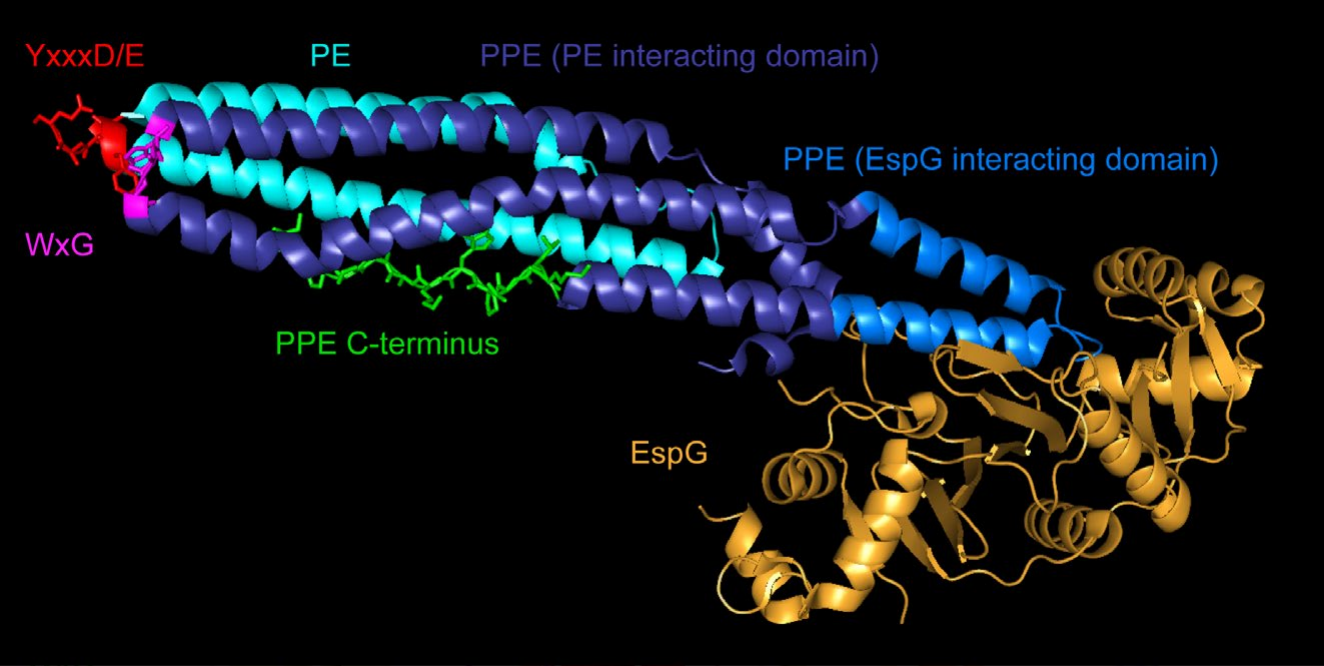
Structure of the EspG5‐PPE41‐PE25 complex (4KXR). The EspG chaperone (Gold) binds to the EspG‐binding domain of the PPE protein (Light blue), thereby conferring TypeVII secretion system specificity (Daleke *et al*., 2012b; Korotkova *et al*., 2014; Phan *et al*., 2017). The rest of the PPE‐protein interacts with its PE partner (Teal) via hydrophobic interactions. The conserved WxG residues of the PPE (Pink) and YxxxD/E of the PE (Red) are closely associated and may form a composite TypeVII secretion signal. The C‐terminus of the PPE (Green) extends toward the C‐terminus of the PE (Red), suggesting that PE/PPE protein pairs with C‐terminal extensions may have further PE/PPE interactions between those specific domains.

The N‐termini of all PE proteins are highly conserved and consist of approximately 100 amino acids that form a helix‐turn‐helix structure (PFAM: PF00934 [Finn *et al.*, 2014]) (Cole *et al.*, 1998; Gey van Pittius *et al.*, 2006; Strong *et al.*, 2006). The pro‐glu residues responsible for naming the protein are generally situated in the ten N‐terminal amino acids, while the C‐terminus of the PE‐domain contains the conserved YxxxD/E TypeVII secretion signal (Fig. 1) (Champion *et al.*, 2006; Daleke *et al.*, 2012a). This secretion signal is required for secretion of these proteins and may be involved in substrate recognition by the TypeVII secretion systems (Champion *et al.*, 2006; Daleke *et al.*, 2012a; Rosenberg *et al.*, 2015; Solomonson *et al.*, 2015). Similarly, the PPE proteins contain highly conserved features. The PPE domain (PFAM: PF00823 [Finn *et al.*, 2014]) is typically around 180 amino acids long and contains the pro‐pro‐glu motif near its N‐terminus. In some cases, both PE and PPE amino acid sequences may not be fully conserved, which should therefore not be used as the sole criterion to recognize PPE proteins (Gey van Pittius *et al.*, 2006). A conserved WxG motif is found between the second and third alpha helix of all PPE proteins, but PPE proteins do not contain the YxxxD/E secretion signal (Fig. 1; Daleke *et al.*, 2012a; Phan and Houben, 2018).

In all studies with sufficient biochemical characterization, PE and PPE proteins were found to be secreted as heterodimers. The PPE protein binds to its cognate PE protein via hydrophobic interactions with the three N‐terminal alpha‐helices of the PPE (Strong *et al.*, 2006; Ekiert and Cox, 2014; Korotkova *et al.*, 2014; Chen *et al.*, 2017). This heterodimeric structure places the conserved WxG of the PPE protein and the YxxxD/E sequence of the PE protein in close proximity and these sequences likely form a composite recognition structure for TypeVII secretion (Fig. 1; Daleke *et al.*, 2012a; Poulsen *et al.*, 2014; Ates *et al.*, 2016a). This PE and PPE complex structure highly resembles the structure of the heterodimers formed by Esx proteins, such as EsxA‐EsxB (ESAT‐6/CFP10) (Renshaw *et al.*, 2005; Poulsen *et al.*, 2014), or even the *Staphylococcus aureus* EsxA or EsxC homodimers (Sundaramoorthy *et al.*, 2008; Anderson *et al.*, 2013; Ates *et al.*, 2016a). The structure of PE and PPE proteins is also highly reminiscent of that of a number of ESX‐1 secretion‐associated proteins (esp) proteins such as the EspA/C and EspE/F heterodimers and EspB (Bitter *et al.*, 2009; Solomonson *et al.*, 2015; Lou *et al.*, 2017; Phan *et al.*, 2018; Phan and Houben, 2018). Together, these data suggest that the helix‐bundle structure and composite secretion signal are among the defining features of PE and PPE heterodimers and other TypeVII‐associated substrates (Daleke *et al.*, 2012a).

A unique characteristic of the Esp and PPE proteins is that helices 4 and 5 do not directly interact with the PE secretion partner. Instead, they bind to cytosolic EspG chaperones and thereby confer system specificity to these substrates (Fig. 1; Daleke *et al.*, 2012b; Ekiert and Cox, 2014; Korotkova *et al.*, 2014; Phan *et al.*, 2017; Phan and Houben, 2018). Intriguingly, exchanging the EspG interacting domain of the ESX‐1 secreted PPE68_1 with that of the ESX‐5 substrate PPE18 was sufficient to reroute this protein from ESX‐1 to ESX‐5 in *M. marinum* (Phan *et al.*, 2017). Therefore, the sequence of the EspG‐binding domain of the PPE protein may be sufficient to predict system specificity of PPE proteins, although experimental validation of this hypothesis remains incomplete beyond the ESX‐1 and ESX‐5 systems (Ekiert and Cox, 2014; Korotkova *et al.*, 2014; Chen *et al.*, 2017; Phan and Houben, 2018). Many of these studies have been performed in the model organism *M. marinum*, but most results have been verified in *M. tuberculosis*. However, some mechanistic differences also remain between *M. marinum* and *M. tuberculosis*, which is understandable when considering the different host ranges and physiological characteristics such as optimal growth temperature. For instance, EspG_5_ was found to be required for all ESX‐5‐dependent phenotypes in *M. marinum* (Abdallah *et al.*, 2009), but dispensable for ESX‐5‐mediated secretion of selected ESX‐5 substrates or virulence in *M. tuberculosis* (Bottai *et al.*, 2012). Similarly, while essentiality of the ESX‐5 system of *M. marinum* can be complemented by introduction of *M. tuberculosis esx‐5*, this complementation did not fully restore PE‐PPE secretion (Ates *et al.*, 2015). A new report pinpoints this species specificity to EccC_5_ and specifically to the linker 2 domain, which is suggested to mediate secretion of specific PE and PPE proteins secreted by ESX‐5 (Bunduc *et al.*, 2019). This illustrates that even when differences between model organisms and *M. tuberculosis* are found, such species specificity can be used to gain more insight into the mechanisms of TypeVII secretion (Bunduc *et al.*, 2019).

More insight into PE and PPE proteins may be obtained by comparing them with other closely related TypeVII secretion substrates. Recent studies have postulated that the EspB protein and the EspA/C heterodimer may multimerize and may form large pore‐ or needle‐like structures that could span the mycobacterial outer membrane (Solomonson *et al.*, 2015; Lou *et al.*, 2017). It remains to be determined if similar structures are also formed by PE and PPE dimers to carry out channel‐like functions, which would align with other experimental data discussed below. An alternative hypothesis is that TypeVII secretion substrates function as autotransporter‐like molecules, which require PE and PPE heteromultimeric complexes for transport over the mycobacterial outer membrane. The structure of the *M. xenopi* ESX‐5 membrane complex was recently visualized by negative stain cryo‐electron microscopy and revealed that the conserved membrane components (EccBCDE) are all present in the inner membrane and their dimensions do not allow for a one‐step secretion process over both the inner and outer mycobacterial membrane (Beckham *et al.*, 2017). Even more recently, high‐resolution cryo‐electron microscopy structures have been resolved of the *M. smegmatis* ESX‐3 secretion systems (Famelis *et al.*, 2019; Poweleit *et al.*, 2019). These structures mostly confirm what was found for *M. xenopi* ESX‐5, but do show in more detail that EccB_3_ protrudes into the periplasmic space, where it could perhaps interact with other periplasmic, or outer membrane, proteins. Yet, no outer membrane components for TypeVII secretion machinery have been identified to date. It is tempting to speculate that such a mechanism for transport over the outer membrane would be encoded in the *esx*‐loci together with the inner membrane components. This function is not performed by other widely present genes in the *esx*‐loci, such as the inner membrane‐localized mycosin proteases (MycP), the EspG chaperones or the cytosolic EccA ATPases, which have clearly characterized other functions and localization (Daleke *et al.*, 2012b; Wagner *et al.*, 2014; Van Winden *et al.*, 2016). There are therefore no clear candidates encoded in the *esx*‐loci that could be involved in outer membrane translocation, apart from the ESX/PE/PPE/Esp substrates. This suggests that either these substrates, or an individually encoded locus, is responsible for outer membrane transport of TypeVII secretion substrates. Interestingly, TypeVII‐like secretion systems have also been identified in monoderm gram‐positive bacteria such as *S. aureus* and *S. intermedius*. These secretion systems secrete bacterial toxins belonging to the WxG (PF06013) and LxG (PF04740) family of TypeVII secretion substrates (Finn *et al.*, 2014; Cao *et al.*, 2016; Whitney *et al.*, 2017). However, since no PE and PPE orthologues have been found in these monoderm bacteria it is tempting to speculate on a role in the secretion process for the PE and PPE proteins, but this is currently nothing more than a highly speculative hypothesis. The fundamental question of how TypeVII secretion substrates cross the mycobacterial outer membrane is clearly of great interest and identification of such mechanisms could even provide leads toward the discovery of novel antimycobacterial compounds (Rybniker *et al.*, 2014).

In summary, PE and PPE proteins share general features in the form of a helix‐bundle structure that allows PE and PPE proteins to interact. This interaction creates a composite secretion signal of the WxG sequence on the PPE and YxxxD/E on the PE, which are likely important for substrate recognition. The PPE proteins contain a two‐helix domain that interacts with EspG chaperones to confer system specificity.

## Subclassification of PE and PPE proteins

Most of the diversity and arguably the most interesting features of the PE and PPE proteins can be found in the highly variable C‐terminal domains. These C‐terminal domains can also be used to classify the PE and PPE proteins in different subgroups, which may greatly aid researchers to formulate testable research hypotheses and provide functional or evolutionary insight (Gordon *et al.*, 1999; Brennan and Delogu, 2002; Gey van Pittius *et al.*, 2006; Delogu *et al.*, 2017).

The largest subgroup that can be distinguished are the polymorphic gc‐rich sequences (PGRS), which define 65 of the 99 PE proteins present in the *M. tuberculosis* reference genome H37Rv (Supplemental Table 1; Poulet and Cole, 1995; Cole *et al.*, 1998; Gey van Pittius *et al.*, 2006). With *gc*‐contents reaching over 80% in some cases, hydrophobic glycine‐rich repeats and large sizes (up to 1400 amino acids), the PE_PGRS subgroup is the most difficult to study. The rest of the PE proteins cannot be reliably subgrouped in further functional categories based on C‐terminal sequences. However, more information can be obtained from the sequences of the PE domains and their genetic environment (Supplemental Table 1). Gey van Pittius *et al*. have used sequence phylogeny of the PE proteins to thus classify these into five sublineages (Gey van Pittius *et al.*, 2006). The ancestral *pe*‐genes encoded within ESX‐1 (PE35) and ESX‐3 (PE5) are defined as sublineage I and II, respectively. Sublineage III may be the most loosely defined sublineage and includes the *esx2*‐localized PE36 as well as the ESX‐5 substrate PE25. Sublineage IV is a group of ESX‐5 secreted PE proteins, whose genes are often transcribed as bicistronic transcripts together with PPE‐SVP’s (discussed below) (Gey Van Pittius *et al.*, 2001; 2006). Sublineage V includes all PE_PGRS proteins, but also a number of proteins with unique C‐terminal domains, including putative hydrolases, lipases and cutinases, with LipY as a best‐known example (Supplemental Table 1; Deb *et al.*, 2006; Daleke *et al.*, 2011; Burggraaf *et al.*, 2019). A number of other PE proteins are putative hydrolases (PE1, PE3, PE4 & PE16) based on structural homology searches by Phyre2 software (Three closest hits with 99% confidence: PDB 3AJA, 1QOZ & 3HC7; Berman *et al.*, 2000; Payne *et al.*, 2009). This hydrolase‐like fold is annotated as the PF08237 domain, which was originally named the ‘PE/PPE domain’ by Adindla and Guruprasad (Adindla and Guruprasad, 2003; Gey van Pittius *et al.*, 2006; Kelley and Sternberg, 2009; Finn *et al.*, 2014). The PFAM08237 domain is also found in the PPE proteins PPE28, PPE42 and PPE63, as well as non‐TypeVII substrate proteins (Gey van Pittius *et al.*, 2006; Finn *et al.*, 2014), indicating that these putative enzymatic domains may have become TypeVII substrates by genetic recombinations with *pe and ppe* genes. It should be noted that PE2, which also contains PFAM08237, is likely not an actual PE protein, since it does not contain any of the PE, YxxxD/E or helix‐turn‐helix hallmarks (Supplemental Table 1; Cole *et al.*, 1998; Kelley and Sternberg, 2009). Interestingly, the lipase LipY is a PE protein in *M. tuberculosis*, while it is a PPE protein in *M. marinum*. Together, the identification of these ESX‐5‐secreted lipases and putative hydrolases and cutinases suggests that such TypeVII‐secreted PE and PPE proteins may be involved in the biogenesis and homeostasis of the cell envelope, or in the uptake of lipid‐based nutrients (Ates *et al.*, 2015; Bosserman and Champion, 2017).

The PPE protein can be subdivided in even clearer subgroups (Fig. 3). Besides the ESX‐1‐specific (PPE68) and ESX‐2‐specific (PPE69) PPE’s, the first clearly defined subgroup is that of the PPE‐PPW proteins. These can be distinguished by a PxxPxxW amino acid motif generally placed between 10 and 30 amino acids from their C‐terminus. *M. tuberculosis* H37Rv contains 10 PPW proteins (Supplemental Table 2), although PPE48 and PPE67 are truncated proteins that may not be functional (Cole *et al.*, 1998; Gey van Pittius *et al.*, 2006; Kapopoulou *et al.*, 2011).

With 26 members, the largest subgroup of PPE proteins are the PPE‐SVP proteins, which are named for the conserved SVP (Serine‐Valine‐Proline) amino acid sequence present in their C‐terminal domain (PFAM: PF12484). PPE50 is likely a truncated protein and PPE9 is not a true PPE‐SVP, because it consists of only a PPE domain of 180 amino acids, but was classified as a PPE‐SVP protein based on phylogenetic analysis (Gey van Pittius *et al.*, 2006). The remaining 24 PPE's are all between 350 and 468 amino acids in size.

Finally, the most recently evolved subgroup of PPE proteins is that of the Major Polymorphic Tandem Repeat (MPTR)‐containing PPE‐MPTR proteins (Hermans *et al.*, 1992; Cole *et al.*, 1998; Gey van Pittius *et al.*, 2006). The MPTR‐repeat consists of repeats that vary around a NxGxGNxG motif. While some PPE‐MPTR proteins are moderately sized, members of this group have unusually large sizes for secreted proteins, ranging up to 3716 amino acids (PPE56; Cole *et al.*, 1998; Gey van Pittius *et al.*, 2006; Kapopoulou *et al.*, 2011).

## Secretion and functions of specific PE and PPE protein subgroups

**Figure 2:**
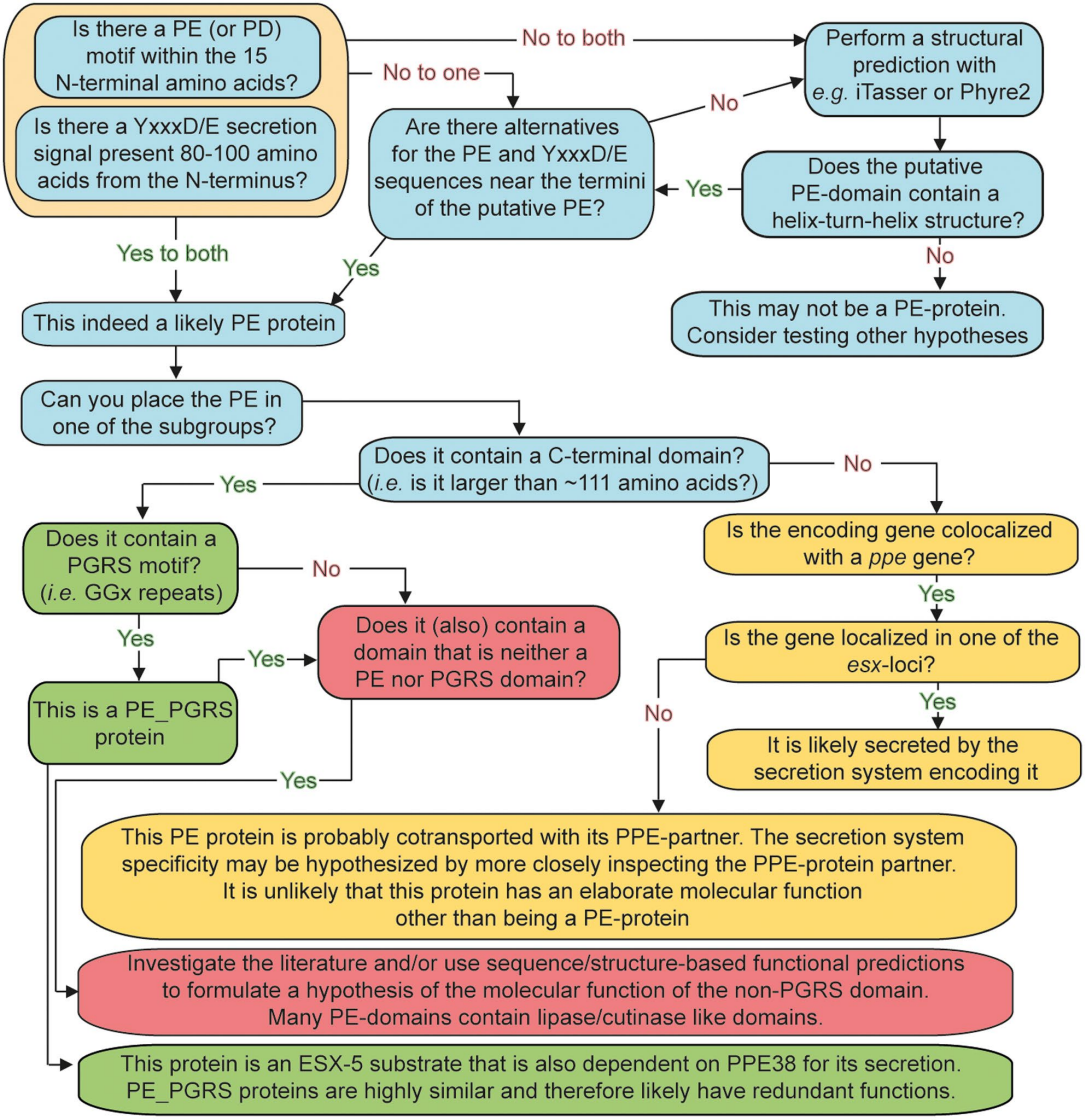
A proposed flow chart to aid researchers to identify and classify PE‐proteins and to formulate testable hypotheses. When interested in a PE gene or protein, a first step can be to verify that it is indeed a functional PE protein, by investigating presence of the defining characteristics (Start at the left top of the flow chart). A following step is to classify the PE‐protein based on its C‐terminal domains (or lack thereof) and genomic localization.

**Figure 3:**
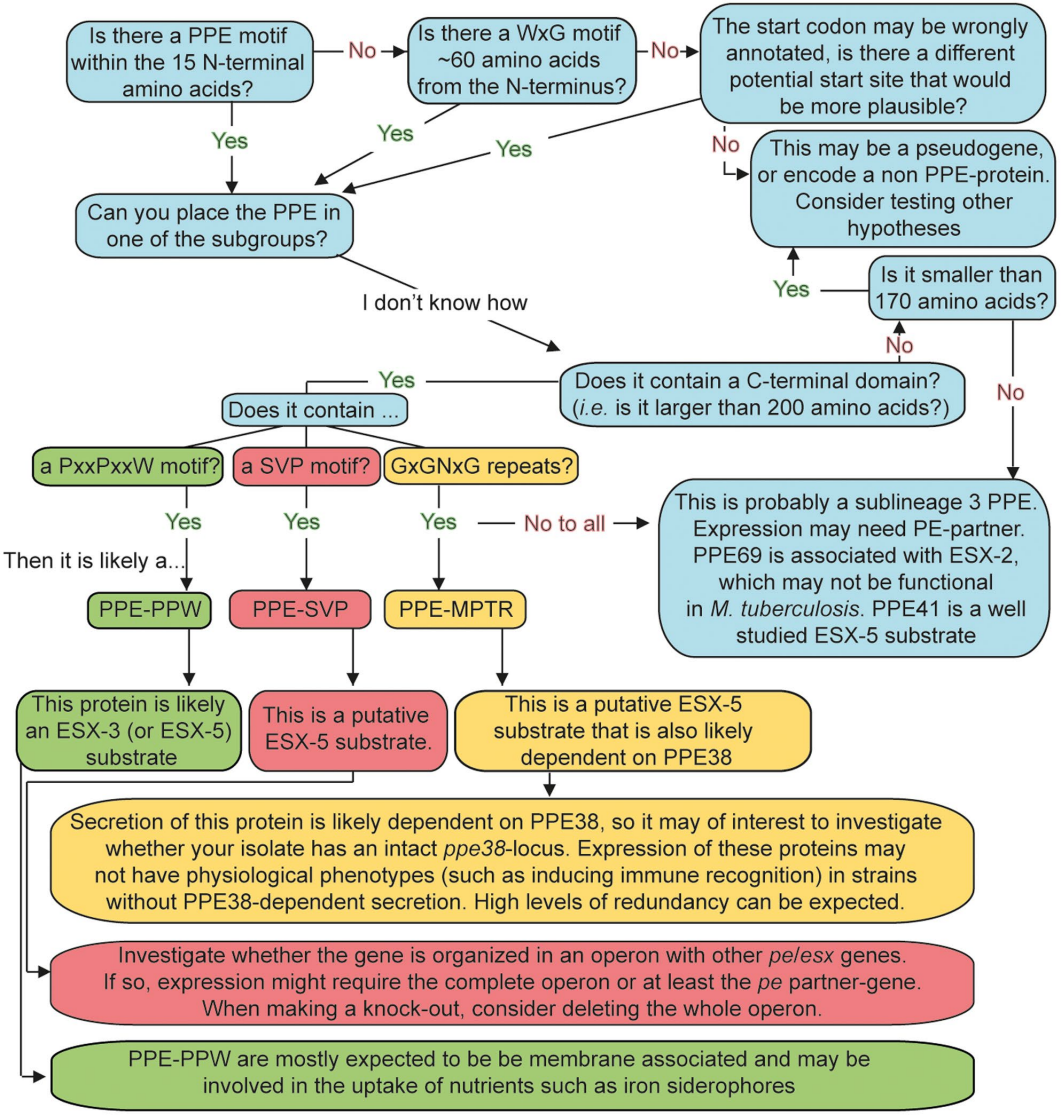
A proposed flow chart to aid researchers to identify and classify PPE proteins in order to formulate testable hypotheses. When interested in a PPE gene or protein, a first step can be to verify it is indeed a functional PPE protein, by investigating presence of the defining characteristics (Start at the left top of the flow chart). A following step is to classify the PPE‐protein as a PPE‐PPW (Green), PPE‐SVP (Red), PPE‐MPTR (Yellow), or other (blue), based on its C‐terminal domains (or lack thereof).

Subdividing PE and PPE proteins as described above (Figs 2 and 3) can help researchers to systematically formulate hypotheses and approaches for experiments. First of all, while generally all *pe and ppe* genes are excluded from bioinformatic datasets, this approach is overly stringent in most cases. Even short‐read sequencing techniques can reliably map almost all *pe and ppe* genes thanks to paired‐end technologies and increased read lengths, if only *pe_pgrs* and *ppe‐mptr* genes/transcripts are excluded (Miran and Farhat – personal communication, Holt *et al.*, 2018; Ates *et al.*, 2018a; Walter *et al.*, 2019). Furthermore, knowing the subgroup of a PE/PPE can be an excellent starting point to hypothesize the most‐likely route of secretion and may even suggest functions, or redundancy. It should be noted that even within the subgroups discussed below, individual proteins may behave markedly different from the other members of their groups. Therefore, the subgrouping may help to provide testable hypotheses, but these should always be validated experimentally before strong conclusions can be drawn.

The best‐studied member of the PPE‐PPW proteins is PPE4, which is genetically encoded within the ESX‐3 genetic locus and is likely secreted by this secretion system, together with its secretion partner PE5 (Korotkova *et al.*, 2014; Tufariello *et al.*, 2016). These proteins are important for mycobactin‐mediated iron acquisition (Siegrist *et al.*, 2009; Tufariello *et al.*, 2016). The PPE‐PPW PPE20 and its cognate partner PE15 were experimentally identified as ESX‐3 substrates (Tufariello *et al.*, 2016). Two other PPW proteins, PPE36 and PPE37, are also described to play a role in iron homeostasis through functions in heme‐iron acquisition (Mitra *et al.*, 2017; Tullius *et al.*, 2019). Together with sequence analysis of the EspG‐binding domain, these data render the hypothesis that PPE‐PPW proteins may be generally dependent on ESX‐3, rather than ESX‐5 for their translocation, increasingly likely (Abdallah *et al.*, 2009; Korotkova *et al.*, 2014; Tufariello *et al.*, 2016).

There is strong experimental evidence that all PPE‐SVP proteins are secreted by ESX‐5 (Abdallah *et al.*, 2009; Bottai *et al.*, 2012; Sayes *et al.*, 2012; Ates *et al.*, 2015). PPE‐SVP proteins are often transcribed as bicistronic transcripts with a sublineage IV PE‐protein and in many cases also two Esx‐proteins (Gey Van Pittius *et al.*, 2001; 2006). These operonic clusters are likely to be the first *pe* and *ppe* genes duplicated from the *esx* genetic loci after introduction of the ESX‐5 system into the most‐recent common ancestor of the slow‐growing mycobacteria (Gey van Pittius *et al.*, 2006; Fishbein *et al.*, 2015; Dumas *et al.*, 2016). The PE and PPE proteins encoded by such operons are thought to be exclusively secreted as PE and PPE heterodimers that may not be promiscuous to other secretion partners (Ekiert and Cox, 2014; Korotkova *et al.*, 2014; Chen *et al.*, 2017; Phan *et al.*, 2017; Phan and Houben, 2018). A notable example of such operonic organization is the PE8‐PPE15‐EsxI‐EsxJ operon, which was shown to facilitate protein secretion of other TypeVII and non‐TypeVII substrates (Shah *et al.*, 2015). Similarly, the PE13‐PPE18‐EsxK‐EsxL is another well‐studied operon, of which PPE18 represents an important antigen component of the M72/AS01E subunit vaccine candidate (Meeren *et al.*, 2018). Another important PPE‐SVP protein is PPE38, which is required for the secretion of all detected PE_PGRS and PPE‐MPTR proteins, which will be discussed in detail below (Ates *et al.*, 2018b; 2018c).

The PPE‐MPTR proteins are secreted by ESX‐5 (Abdallah *et al.*, 2009) and are furthermore dependent on PPE38 for their secretion (Ates *et al.*, 2018b). Loss‐of‐function mutations in the *ppe38* locus render many clinical *M. tuberculosis* isolates and other MTBC members unable to secrete these proteins and may therefore provide information on these groups as a whole (Ates *et al.*, 2018b; 2018c). An important function in capsule integrity of *M. marinum* was shown for the ancestral PPE‐MPTR protein PPE10 (Gey van Pittius *et al.*, 2006; Ates *et al.*, 2016b), although this phenotype was not apparent in *M. tuberculosis* (Ates *et al.*, 2018c). PPE62 is reported to be a surface accessible heme‐binding protein, which is not strictly required for virulence of *M. tuberculosis* (Mitra *et al.*, 2017). The PPE‐MPTR protein PPE42 is an immunogenic protein that is part of the ID93 subunit vaccine candidate (Bertholet *et al.*, 2008; 2010; Baldwin *et al.*, 2015).

The secretion of all PE‐proteins with available data, except those encoded within the ESX‐1, ESX‐2 and ESX‐3 loci and those associated with PPE‐PPW proteins, is thought to be mediated by the ESX‐5 secretion system (Abdallah *et al.*, 2009; Sayes *et al.*, 2012, 2016; 2018; Houben *et al.*, 2012b; Ates *et al.*, 2015; 2018c). The *esx5*‐encoded PE19 is of general interest since it has been postulated to play a role in phosphate homeostasis and stress resistance (Ramakrishnan *et al.*, 2015), and it is part of the *ppe25*‐*pe19* gene cluster that attenuates *M. tuberculosis* when deleted in a preclinical vaccine candidate (Bottai *et al.*, 2012; Sayes *et al.*, 2012).

A significant amount of research has been dedicated to the largest group of the PE_PGRS proteins, in part because of the availability of a monoclonal antibody that recognizes the repetitive PGRS domain (Abdallah *et al.*, 2009). Similar to the PPE‐MPTR proteins, PE_PGRS are not only dependent on ESX‐5 for their secretion, but also on the presence of a functional PPE38 protein (Ates *et al.*, 2018b). It is not yet known whether this is true only for PE_PGRS proteins, or also for other sublineage‐V PE‐proteins, that is, PPE38 required for secretion of the PGRS/MPTR domains specifically? Or, is it required for all sublineage‐V PE and PPE’s by for instance functioning as the partner in heterodimeric secretion complexes that can interact with the chaperone EspG_5_ (Daleke *et al.*, 2012b; Phan *et al.*, 2017)? While this latter possibility was the major hypothesis upon identification of PPE38 as an essential factor for PE_PGRS secretion, it is hard to reconcile this hypothesis with the finding that PPE38 is also required for PPE‐MPTR secretion (Ates *et al.*, 2018b).

**Figure 4:**
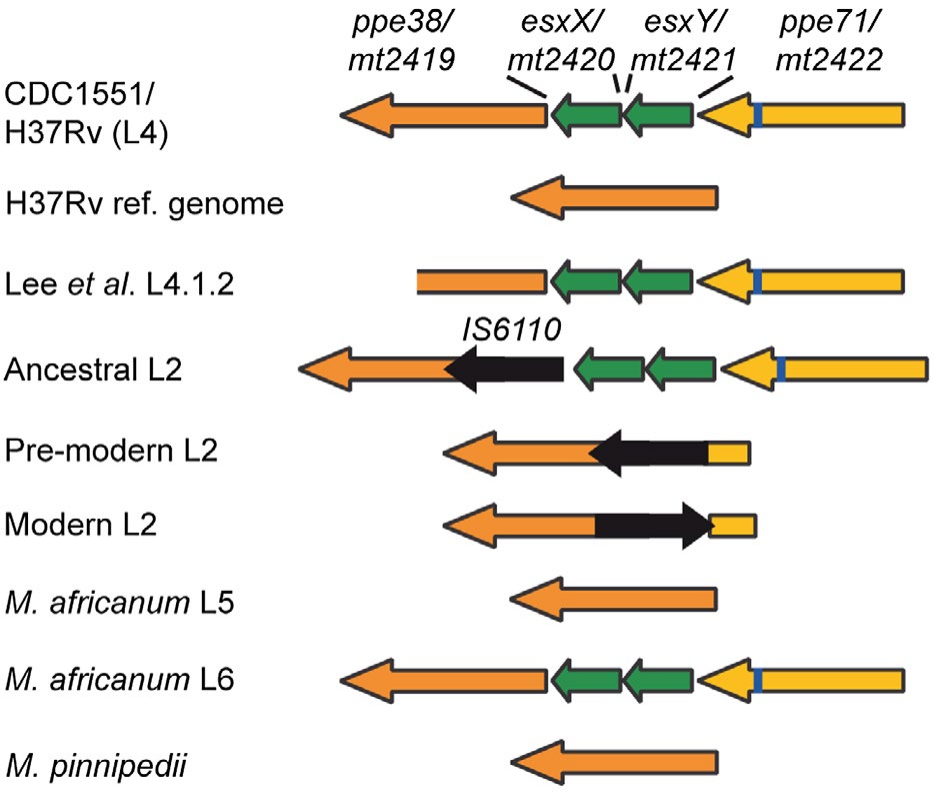
A selection of existing configurations of the *ppe38‐71* locus. Gene numbers correspond to the *M. tuberculosis* CDC1551 reference genome. *ppe38* (orange) and *ppe71* (yellow) are genetically identical except for a 21bp deletion (blue) in *ppe71*. The two *esx*‐genes (green) have been named *esxX* and *esxY* based on standardized nomenclature (Bitter *et al*., 2009; Ates *et al*., 2018b). The most common configuration of the locus is depicted at the top (McEvoy *et al*., 2009a). The H37Rv reference genome (second line) is misannotated as having only one copy of *ppe38*, but multiple H37Rv strains were shown to possess the CDC1551‐like locus (Box 1; McEvoy *et al*., 2009a; Ates *et al*., 2018b). However, the configuration of a single copy of either *ppe38*, or *ppe71*, does occur due to recombination in diverse clinical isolates and is referred to as RvD7 (McEvoy *et al*., 2009a). An RD5‐like deletion that truncates *ppe38*, but keeps *ppe71* intact, was found in certain L4 isolates. This polymorphism did not negatively affect PPE38‐dependent secretion (Lee *et al*., 2015; Ates *et al*., 2018c). Similarly, in an ancestral L2‐isolate (SAWC_2088) an IS6110 (black) insertion truncation *ppe38* does not negatively affect PPE‐38‐dependent secretion. Subsequent recombination that occurred at the branching point of the modern L2‐lineages, have also truncated *ppe71* and deleted *esxXY*, resulting in a loss of PPE38‐dependent secretion (McEvoy *et al*., 2009a; Ates *et al*., 2018b). It should be emphasized that many more configurations of the *ppe38‐71* locus, including different RD5‐like deletions have been more thoroughly described in McEvoy *et al*., 2009b (McEvoy *et al*., 2009a).

Many virulent *M. tuberculosis* strains, such as the modern L2 lineage, do not secrete PE_PGRS and PPE‐MPTR proteins, because of loss‐of‐function mutations in the *ppe38* genetic locus (Ates *et al.*, 2018b; 2018c; Fig. 4). From these findings it can be postulated that secretion of neither PE_PGRS nor PPE‐MPTR proteins is essential for virulence of *M. tuberculosis* in humans. In fact, loss of secretion of these proteins actually increases virulence of *M. tuberculosis* (Ates *et al.*, 2018b). The latter observation is bolstered by the finding that a *M. marinum* mutant with a disrupted *espG_5_* gene is hypervirulent in adult zebrafish (Weerdenburg *et al.*, 2012). Intriguingly, the increased virulence of *M. tuberculosis ppe38* mutants in mice is only apparent in later stages of infection (3–6 weeks post infection) and *M. marinum espG_5_::tn* was similarly not hypervirulent in zebrafish embryos (Weerdenburg *et al.*, 2012; Ates *et al.*, 2018b). Therefore, this hypervirulence phenotype could be related to interactions with the host’s adaptive immunity or bacterial adaptation at later stages of infection. Importantly, genetic perturbations that completely abrogate ESX‐5 secretion are lethal to slow growing mycobacteria (Di Luca *et al.*, 2012; Ates *et al.*, 2015). The absence of *in vitro* growth defects and the increased virulence of *M. tuberculosis* with *ppe38 *deletions suggests that substrates that are responsible for the essentiality of ESX‐5 likely do not belong to the PE_PGRS or PPE‐MPTR subgroups (Ates *et al.*, 2018b). Finally, it should be noted that only one particular transposon insertion site in the 5′ of *espG_5_* was tolerated by *M. marinum*. Likely, because this results in a truncated EspG_5_ that allows some residual ESX‐5 secretion (Ates *et al.*, 2015). Full deletions, or transposon insertion in the middle of *espG_5_*, were only possible in the context of expression of MspA, which circumvents essentiality of ESX‐5 in *M. marinum* (Ates *et al.*, 2015). Care should be taken to extrapolate these findings to *M. tuberculosis*, because EspG_5_ may have a more redundant role in *M. tuberculosis* compared to *M. marinum* (Bottai *et al.*, 2012).

Confusingly, the recent data reporting increased virulence in strains that do not secrete PE_PGRS and PPE‐MPTR proteins are in stark contrast with numerous previous studies reporting PE_PGRS as important virulence factors that perform a plethora of functions related to host–pathogen interaction. These proposed functions include inhibition of apoptosis or antigen presentation, induction of host‐cell death and modulation of immune responses by binding to Toll‐like receptors (reviewed in: Sampson, 2011; Fishbein *et al.*, 2015; Delogu *et al.*, 2017). Many studies on PE_PGRS proteins used expression of these proteins in *M. smegmatis* as a model, which usually lead to increased persistence of the bacteria in macrophage or mouse models. It is hard to envisage the biological relevance of this model, since *M. smegmatis* does not possess the appropriate chaperones, secretion partners or secretion system to express PE_PGRS proteins in soluble form and secrete them. A promising new approach that could overcome this obstacle is the recent success in expression of a functional ESX‐5 system from *M. xenopi* in *M. smegmatis*, which enabled resolution of the first TypeVII secretion system structure by cryo‐electron microscopy. This heterologously expressed ESX‐5 system was able to facilitate secretion of the PPE‐SVP protein PPE18 as well as EsxN in *M. smegmatis* (Beckham *et al.*, 2017). No secretion of PE_PGRS proteins has been reported in this model, which is also not expected to occur, since no functional PPE38 is present in this system. However, such a model has great potential to build a ‘bottom‐up’ model of the secretion requirements of different classes of PE and PPE proteins.

Other studies, performed in arguably more appropriate models, such as *M. tuberculosis* or *M. marinum*, have also reported roles for PE_PGRS proteins as virulence factors. For instance, Saini *et al*. reported that PE_PGRS47 suppresses autophagy and impairs antigen presentation (Saini *et al.*, 2016). Similarly, PE_PGRS30 was reported to be important for *M. tuberculosis* virulence in mice, especially in chronic infection (Iantomasi *et al.*, 2012). Another interesting study identified the *M. marinum*‐specific PE_PGRS protein MMAR_0242 as an important virulence factor that was dependent on the unique C‐terminal domain and not the PGRS domain itself (Singh *et al.*, 2016). These high‐quality studies represent only a fraction of the available literature on the various roles of PE_PGRS (and also PPE‐MPTR) proteins in virulence, which is reviewed more extensively by Delogu *et al.*, (2017), Fishbein *et al.* (2015) and Sampson (2011). In contrast with the available literature, we have not found differences in dendritic cell activation, antigen presentation or cytokine production in response to short‐term infection with *M. tuberculosis* or *M. bovis* BCG strains with or without functional PPE38‐dependent PE_PGRS/PPE‐MPTR secretion (Ates *et al.*, 2018c). This may however be dependent on the dynamics of infections in different infection models, which may vary in their response depending on host species and cell type utilized. Furthermore, the finding that many virulent clinical isolates do not secrete these proteins does not directly suggest an important role as virulence factors at the host–pathogen interface for these proteins (Ates *et al.*, 2018a; 2018b; 2018c). However, a major difference between these studies is the contrast between blocking secretion of the complete PE_PGRS/PPE‐MPTR groups, while leaving expression of the proteins untouched, versus creating genetic knock‐outs of single *pe_pgrs* genes. These conflicting data from different approaches could suggest roles for the PE_PGRS proteins that are not dependent on their protein secretion and are therefore likely independent of their direct interaction with the host. Alternatively, deletion of single PE_PGRS proteins could have indirect effects on other ESX‐5 substrates that may explain certain virulence effects. Together, these exciting and seemingly contradictory findings create a puzzling tension in the field that begs for a unifying explanation which integrates these findings. Together, recent work has provided new roads forward that have the potential to finally bring more insight into the actual function of PE_PGRS and PPE‐MPTR proteins (Ates *et al.*, 2015; 2018b; Beckham *et al.*, 2017).

## The potential and pitfalls of PE and PPE proteins as vaccine components

PE and PPE proteins contain many immunogenic epitopes and are therefore of great interest for the development of new tuberculosis vaccines (Brennan, 2017). The PGRS domains of PE_PGRS proteins possess only a few predicted immunogenic epitopes, especially compared to their more antigenic PE domains (McEvoy *et al.*, 2012; Copin *et al.*, 2014). However, especially the PPE proteins contain many predicted and experimentally validated antigenic epitopes. Important examples of immunogenic PPE proteins include the PPE‐SVP protein PPE18, which is part of M72/AS01E subunit vaccine candidate (Meeren *et al.*, 2018) and the PPE‐MPTR protein PPE42, which is a part of the subunit vaccine ID93 (Bertholet *et al.*, 2008; 2010; Baldwin *et al.*, 2015). Furthermore, immune responses to PE and PPE proteins also play a large role in designing whole‐cell vaccines. For instance, the preclinical vaccine candidate *M. tuberculosis* Δ*ppe25‐pe19* is attenuated by a partial deletion of the *esx‐5* genetic locus (Bottai *et al.*, 2012; Sayes *et al.*, 2012). Despite this deletion, ESX‐5 secretion remains functional, leading to cross immune recognition of PE and PPE domains from orthologous proteins that are still secreted, thereby minimizing the reduction in the antigenic repertoire of this vaccine strain compared to wildtype (Sayes *et al.*, 2012; 2016). In contrast, this immune recognition is completely lost when blocking ESX‐5 secretion by deleting the ESX‐5 membrane components *eccC_5_* or *eccD_5_* (Sayes *et al.*, 2012; 2016), because secretion through the cognate TypeVII protein secretion system is essential for antigen processing, presentation and recognition by immune cells (Sayes *et al.*, 2018).

Importantly, the BCG vaccine strain does not secrete PE_PGRS and PPE‐MPTR proteins due to deletion of the *ppe38*‐containing RD5‐genetic region in the ancestral *M. bovis* isolate (McEvoy *et al.*, 2009a; Ates *et al.*, 2018c). This secretion defect can likely be generalized to an inability of BCG to induce immune responses against all PE‐PGRS and PPE‐MPTR proteins, which was experimentally confirmed for the ancestral PPE‐MPTR PPE10. Although complementation of BCG with the *ppe38‐71* genetic region restored PE_PGRS secretion and immune recognition of PPE10, no improved (or reduced) protection of this recombinant strain was seen in two different mouse models (Ates *et al.*, 2018c). In spite of this disappointing effect, when faced with a choice it would seem preferable to induce the widest antigenic recognition possible. In that light, attenuated *M. tuberculosis* vaccine candidates, such as the promising MTBVAC (Arbues *et al.*, 2013; Tameris *et al.*, 2019), have the advantage over recombinant BCG that they will usually contain functional PPE38‐dependent secretion.

It is also important to consider the genetic and phenotypic variability within *M. tuberculosis* that can lead to differing PE and PPE recognition. The *pe and ppe* genes are among the most genetically variable genes within the *M. tuberculosis* genome and this variation should therefore be taken into account, especially when subunit vaccines are rolled out in patient populations (McEvoy *et al.*, 2009a; 2012; Copin *et al.*, 2014; Phelan *et al.*, 2016). A prime example for this is the PPE18 component of the M72/AS01E vaccine candidate, for which Homolka *et al*. described 28 non‐synonymous single‐nucleotide polymorphisms, three deletions and one insertion in a panel of 71 MTBC strains (Homolka *et al.*, 2016). Furthermore, expression patterns of PE and PPE proteins may not be constant during infection, which may influence T cell responses and therefore vaccine efficacy (Dheenadhayalan *et al.*, 2006; Goldstone *et al.*, 2009; Moguche *et al.*, 2017).

However, only genetic investigation of epitopes of interest may still underestimate variability, as these proteins need to be secreted to be efficiently processed and presented as antigenic epitopes (Sayes *et al.*, 2018). For instance, the modern L2 (modern Beijing) strains with *ppe38* deletions are not expected to be recognized by T cell or antibody immune responses aimed at epitopes from PE_PGRS or PPE‐MPTR proteins (Ates *et al.*, 2018c). Using such epitopes in subunit vaccines could infer increased protection to strains that also secrete these proteins, and conversely protect less against strains that do not secrete these proteins. This could be doubly disadvantageous, since such strains also seem to be more virulent (Hanekom *et al.*, 2011; Ates *et al.*, 2018b). This could be of particular relevance for PPE42, a PPE‐MPTR protein that is part of the ID93 vaccine candidate, which is currently being tested in phase II clinical trials (Johnson et al., 2008; Phase 2a ID93 + GLA‐SE Vaccine Trial in TB Patients After Treatment Completion – Full Text View – ClinicalTrials.gov, n.d.). Although these scenarios are presently purely hypothetical, it is important to design trials and preclinical investigation in ways that take into account the existing diversity of *M. tuberculosis*. Such measures could include bioinformatic analyses of the expected diversity of epitopes of vaccine candidate proteins within *M. tuberculosis* strains, preferably supplemented by laboratory tests. For instance, immunogenicity and protection of individual subunit components should be tested against multiple divergent strains of *M. tuberculosis*. Finally, epidemiologic monitoring of the circulating strain genotypes after introduction of novel vaccines, as is done for other bacterial vaccines, could help to observe bacterial adaptation once a vaccine is implemented (McIntyre *et al.*, 2012; Azarian *et al.*, 2018).

## Implications of *pe and ppe* gene variation for the evolution of Mycobacteria

The finding that *pe and ppe* genes cover 7–10% of the *M. tuberculosis* genome suggests an important role for these proteins in the evolution of *M. tuberculosis* (Cole *et al.*, 1998; Fishbein *et al.*, 2015). This is further substantiated by the finding that the fast‐growing mycobacteria, which include predominantly saprophytic species, only possess the PE and PPE proteins localized in the *esx*‐loci ESX‐1 and ESX‐3 (Gey van Pittius *et al.*, 2006). Since the slow‐growing mycobacteria, which include most species pathogenic to humans and other animals, have seen remarkable expansion of *pe and ppe* genes, it is tempting to suggest that this expansion has been part of adaptation to an intracellular and pathogenic lifestyle (Gey van Pittius *et al.*, 2006; Fishbein *et al.*, 2015). In contrast, the major mycobacterial human pathogen, *Mycobacterium leprae*, only has a small number of remaining *pe* and *ppe* genes that have not been subject to reductive evolution (Cole *et al.*, 2001; Gey van Pittius *et al.*, 2006). The only exceptions of PE/PPE proteins present in both *M. leprae* and *M. tuberculosis* are PE15‐PPE20 (ML_0538‐9), PE13‐PPE18 (ML_1053 and ML_1182 (an *M. leprae* specific PPE18 paralog)), PE5 (ML_2534c), PPE1 (ML_1991), PPE2 (ML_1828c) and PPE68 (ML_0051c; Cole *et al.*, 1998; 2001; Gey van Pittius *et al.*, 2006). It could be argued that these widely conserved genes may be most relevant to study conserved aspects of mycobacterial pathogenesis and these may include the substrates that make the ESX‐3 and ESX‐5 systems essential for slow‐growing Mycobacteria (Di Luca *et al.*, 2012; Serafini *et al.*, 2013; 2014; Ates *et al.*, 2015; Tufariello *et al.*, 2016).

**Figure 5:**
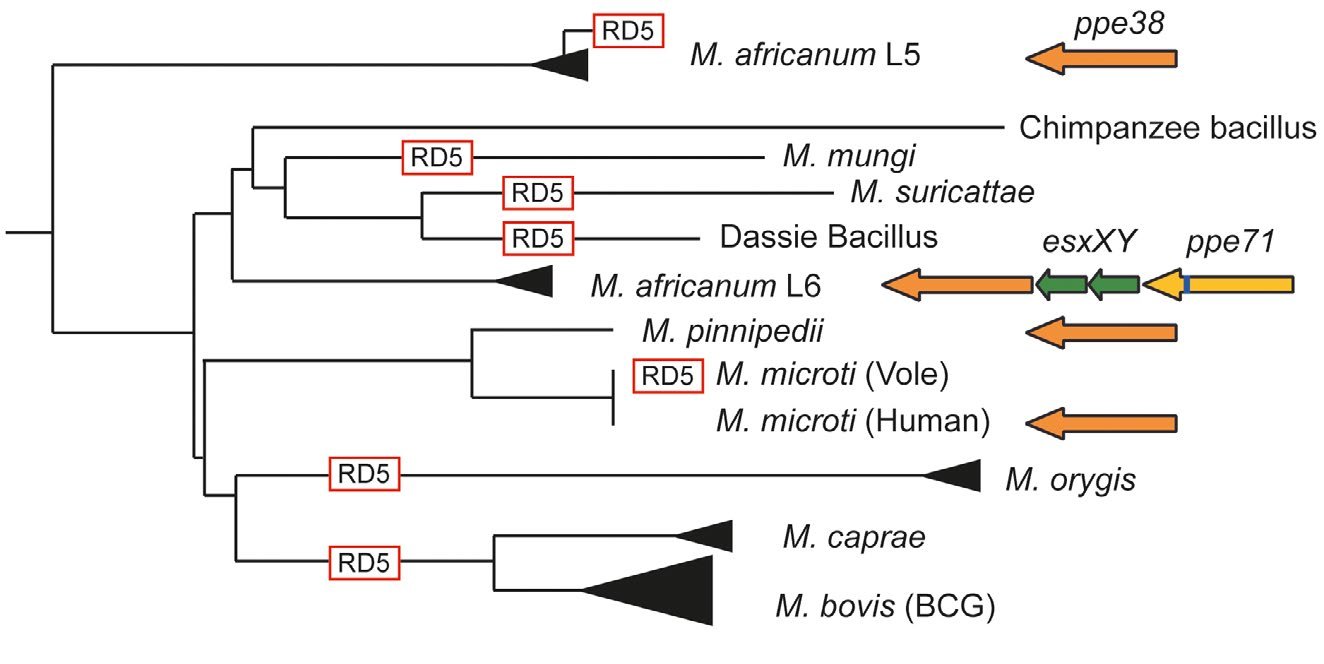
RD5 and *ppe38* polymorphisms in *M. africanum* and the animal‐adapted species of the *M. tuberculosis* complex. The phylogeny is adapted from Brites *et al*. with permission of the author (Brites *et al*., 2018). Independent RD5 deletions, with unique remaining flanking sequences, are depicted in the phylogenetic tree. The organization of the *ppe38* locus in *M. africanum* L5, L6 and *M. microti* and *M. pinnipedii* is indicated with arrows, similar as in Fig. 4. Only one out of 18 M*. africanum* L5 isolates was found to have an RD5‐like deletion (Ates *et al*., 2018a). In a study by Brodin *et al*. three *M. microti* isolates from humans had an intact RD5‐locus, while four strains isolated from voles had RD5‐deletions (Brodin *et al*., 2002).

Neither the PE_PGRS or PPE‐MPTR proteins, nor PPE38 are present as potentially functional ORFs in the *M. leprae* genome, thereby indicating that these proteins are not strictly required for a pathogenic lifestyle in humans. This is concordant with the finding that natural *ppe38* deletion strains that do not secrete PPE‐MPTR and PE_PGRS proteins are highly virulent in humans. However, a role for these proteins in the co‐evolution with human hosts should not be dismissed based on these data. For instance, the majority of pathogenic *M. tuberculosis* strains possess functional copies of PPE38 and corresponding PE_PGRS and PPE‐MPTR secretion. This observation, in combination with that the *ppe38* locus is among the most variable regions of the *M. tuberculosis* genome, suggests that there is an evolutionary pressure to retain PPE38‐dependent secretion (McEvoy *et al.*, 2009a; Ates *et al.*, 2018a; 2018b; 2018c). In contrast, a signature of convergent evolution toward loss of the *ppe38* locus seems especially apparent in the animal adapted isolates of the MTBC (Fig. 5).

These deletions, known as region of difference (RD)5, have occurred independently in *M. bovis*/*M. caprae*, *M. orygis*, *M. suricattae* and *M. mungi*, the Dassie Bacillus and a subset of *M. microti* strains isolated from voles (Brodin *et al.*, 2002; Garnier *et al.*, 2003; Mostowy *et al.*, 2004; Dippenaar *et al.*, 2015; Brites *et al.*, 2018). In fact, the only known animal adapted MTBC members that have retained the RD5 locus are the Chimpanzee bacillus, *M. pinnipedii* and a subset of *M. microti* isolates isolated from humans (Brodin *et al.*, 2002; Coscolla *et al.*, 2013; Brites *et al.*, 2018). Similarly, also the closely related human‐adapted *M. africanum* Lineage six seems to have mostly intact RD5 regions (Fig. 5; Brosch *et al.*, 2002; Brites *et al.*, 2018; Ates *et al.*, 2018a). Together, these evolutionary observations suggest an advantage for the loss of the *ppe38* locus only under specific conditions. An interesting hypothesis is that increased virulence resulting from *ppe38* deletion may confer an advantage under population‐dense situations, allowing fast spread between individuals and relatively quick progression to disease as has been reported for the modern Beijing isolates (Hanekom *et al.*, 2011; Merker *et al.*, 2015; Ates *et al.*, 2018b). However, in most human populations, these traits may actually be disadvantageous and a more chronic disease may be evolutionary favorable (Brites and Gagneux, 2015). This hypothesis would translate well to the animal adapted isolates, where species infecting small animals with relatively short lifespans such as voles, mongooses and meerkats have developed RD5 deletions (Brodin *et al.*, 2002; Dippenaar *et al.*, 2015; Alexander *et al.*, 2016a). It is also particularly intriguing that these mycobacterial species (but also *M. pinnipedii* with an intact RD5) have all obtained independent RD1‐like deletions inactivating the ESX‐1 secretion system, which should theoretically lead to complete attenuation (Brodin *et al.*, 2002; Dippenaar *et al.*, 2015; Alexander *et al.*, 2016a; 2016b). Perhaps the route of transmission in these animals is different from the aerosol transmission between humans and other larger animals. This was suggested for *M. mungi*, which seems to be transmitted via direct contact of mucous tissue in particularly in the nasal and anal areas (Alexander *et al.*, 2016b). The RD1/ESX‐1‐dependent rupture of phagosomal membranes that leads to cellular necrosis and extracellular bacilli may be particularly important for aerosol transmission and may be less important when direct contact between infected and noninfected tissues occurs regularly (van der Wel *et al.*, 2007; Simeone *et al.*, 2012; Houben *et al.*, 2012a; Gröschel *et al.*, 2016; Dallenga *et al.*, 2017). Since the ESX‐1 substrates are highly immunogenic, the potential reduction of virulence could be offset by a reduction in antigenic recognition of the pathogen, especially in this context of reduced importance for transmission. The RD5 deletions in the larger livestock animals such as cattle and goats could potentially be explained by the artificially shortened lifespans due to consumption of these animals. However, it is unclear to what extend this is true for the time in which the most‐recent common ancestor of *M. bovis* and *M. caprae* emerged (Loiseau *et al.*, 2019). In line with the hypothesis that RD5‐deletions make *M. bovis* more virulent, there is experimental evidence that *M. bovis* is more aggressive in cattle compared to *M. tuberculosis* (Whelan *et al.*, 2010). Chimpanzees, seals and sea lions may have longer expected lifespans, and this may therefore have formed an advantage for the chimpanzee bacillus and *M. pinnipedii* to retain *ppe38*. As intriguing as this hypothesis is, it is purely speculative and based on circumstantial evidence. Although the evidence for increased virulence of modern lineage two isolates and *ppe38* deletion mutants is strong in experimental animals (Aguilar *et al.*, 2010; Hanekom *et al.*, 2011; Weerdenburg *et al.*, 2012; Gopal *et al.*, 2014; Ates *et al.*, 2018b), differences are much harder to substantiate in human populations in spite of mounting evidence (Hanekom *et al.*, 2011; Huyen *et al.*, 2013; Merker *et al.*, 2015; Holt *et al.*, 2018). Furthermore, analysis of the full spectrum of genetic variation of *ppe38* locus has been hampered by sequencing and mapping difficulties, due to the misannotated reference genome (Box 1, Fig. 4; McEvoy *et al.*, 2009a).

Box 1 **Methodological difficulties to identify *ppe38* polymorphisms**

The *ppe38*‐containing genomic locus is one of the most variable regions in the *M. tuberculosis* genome (McEvoy *et al.*, 2009a). Unfortunately, a large part of this diversity remains currently uncharted, because of an annotation error in the *M. tuberculosis* H37Rv reference genome (Genbank: AL123456). While the reference genome only contains a single *ppe38* gene, most *M. tuberculosis* genomes contain two copies of the *ppe* (*ppe38* and *ppe71*, which are identical or differ only by a seven‐amino acid insertion) that together flank two esx genes, *esxXY* (Fig. 4; McEvoy *et al.*, 2009a; Ates *et al.*, 2018b). Therefore, it may be more informative to use a different reference genome (e.g. the *M. tuberculosis* CDC1551 reference genome (Genbank: AE000516 – *mt2422‐19*)) when assessing polymorphisms in this region (McEvoy *et al.*, 2009a; Lee *et al.*, 2015; Ates *et al.*, 2018b). Even then, it may be needed to perform PCR amplification and Sanger sequencing, or long‐read sequencing as a verification, because IS6110 insertions may remain difficult to detect (McEvoy *et al.*, 2009a; 2009b).

## The potential of new sequencing techniques and refined sequence analyses

Increased understanding of the actual role of the PE and PPE proteins have played in the evolution of the MTBC may come from more detailed molecular studies identifying the mechanisms of their secretion and their interacting partners. Another approach may be to more accurately map the diversity in *pe and ppe* genes present in the global *M. tuberculosis* isolates with emerging new long read sequencing techniques, but also by more careful analyses. These may include only discarding those *pe* and *ppe* genes which are not reliably sequenced (*e.g.* a curated subset of the *pe_pgrs* and *ppe/mptr* genes), but may also be aided by using more refined reference genomes and mapping algorithms (Maciuca *et al.*, 2016). Refining bioinformatic analysis methods to include *pe and ppe* genes may allow to re‐analyze thousands of published genome datasets and therefore may uncover exciting new information on *M. tuberculosis* (Bradley *et al.*, 2015; The CRyPTIC Consortium and the 100.00 Genomes Project, 2018). For instance, it may be plausible to uncover polymorphisms in *pe* and *ppe* genes that affect antibiotic sensitivity, since PE and PPE proteins are involved in membrane permeability and nutrient uptake (Ates *et al.*, 2015; Tufariello *et al.*, 2016; Mitra *et al.*, 2017). Furthermore, such technical advances are needed to perform more large‐scale studies on the evolutionary pressures exerted on *pe and ppe* genes, such as by studying dN/dS rates in different domains/subgroups of *pe* and *ppe* genes or in predicted antigenic epitope sequences (McEvoy *et al.*, 2012; Copin *et al.*, 2014). A very interesting open question is whether loss of *ppe38* leads to increased/reduced selection pressure on *pe_pgrs*/*ppe‐mptr* genes, since these protein products are then no longer expected to be under selection pressure from immune recognition.

Such approaches may be especially powerful when combined with laboratory and clinical parameters to link genotypes to particular phenotypes. For instance, we recently found a new sublineage of *M. africanum* lineage 5 that was deficient in PE_PGRS and PPE‐MPTR secretion while have a seemingly intact *ppe38* locus (Ates *et al.*, 2018a). This phenotype was not complemented by introduction of the *ppe38‐71* locus, suggesting that another variation is responsible for this phenotype. However, the development of novel tools such as specific antibodies to PPE38 and PPE‐MPTR proteins may be required to gain more biochemical insight in such cases.

## Conclusions

The PE and PPE proteins have puzzled researchers for decades and it is unlikely that their biological roles will be completely elucidated in the coming decade. However, big advances in our knowledge on these proteins have still been achieved recently and ongoing methodological advances may provide further progress. It is of paramount importance to be aware of the associated methodological difficulties when studying these proteins, such as redundancy, sequence complexity, interdependency of substrates and essentiality of secretion systems. Similarly, being aware of the different subgroups of PE/PPE proteins is an essential step toward elucidation of the functions of these proteins, since the most diverse and unique molecular features of individual proteins may be found in the variable C‐terminal domains. Some PE/PPE proteins may be best studies as individuals, for example, single gene knock‐out and complementation studies. In contrast, the PE_PGRS and PPE‐MPTR may in cases be more aptly studied as a subgroup, because of their high similarity and putative redundancy. Such groupings may be especially valuable for bioinformatic studies, since variation and selection pressures may differ remarkably between different subgroups of *pe* and *ppe* genes and grouping all of these genes together may lead to a loss of such signals. In summary, big advances in our knowledge on these proteins have been achieved recently and ongoing methodological advances may provide further progress. If the molecular biology of these intriguing, but sometimes confusing proteins is taken into account, this will lead to a better understanding of the biology of the most successful human pathogen of our times.

## Conflict of interest

The author declares no financial conflict of interest.

## Funding information

Louis S Ates is supported by a PostDoc stipend of the Amsterdam Infection and Immunity Institute and by the Netherlands Organisation for Scientific Research (VIDI grant 91717305 to Jeroen WJ van Heijst).
